# Supplementary material for: Isoform-Specific Role of Akt in Oral Squamous Cell Carcinoma
Source: Biomolecules. 2019 Jun 27;9(7):253. doi: 10.3390/biom9070253 (PMC6681224; doi:10.3390/biom9070253)
Supplement: Supplementary file 1 [file biomolecules-09-00253-s001.pdf]

A

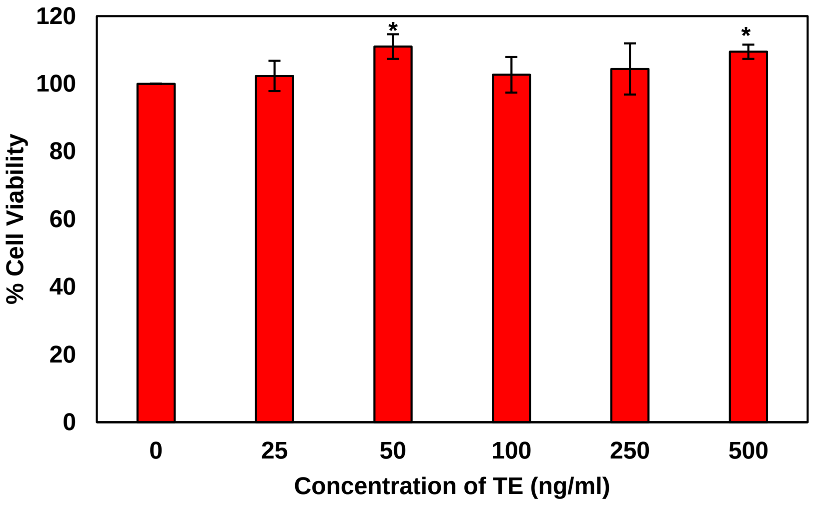

B

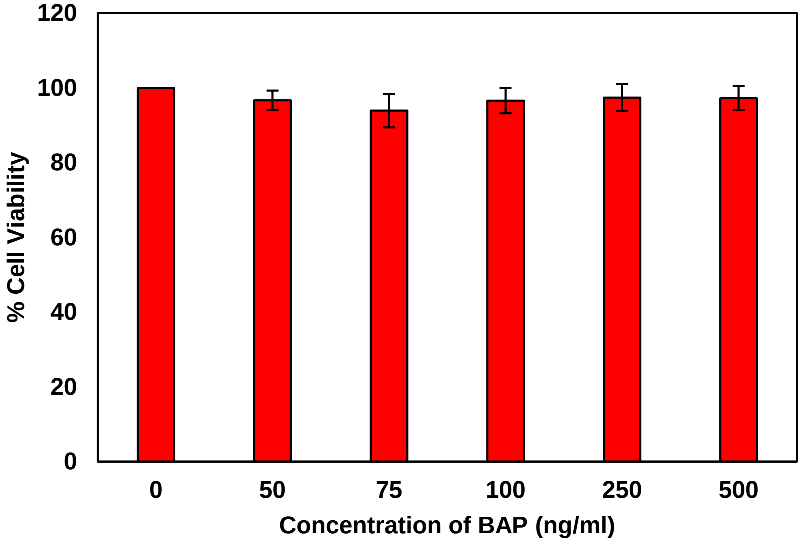

C

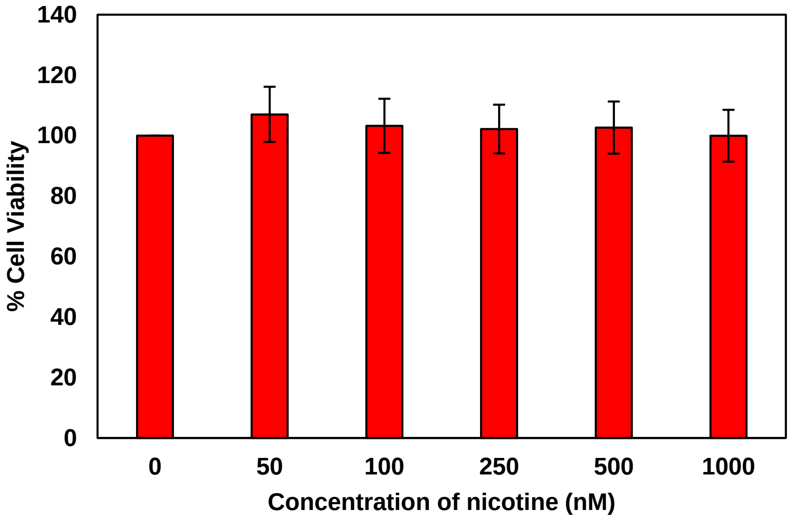

Figure S1. Effect of A; TE, B; BAP, and C; nicotine on the proliferation of SAS cells as analyzed by MTT assay (% Proliferation values shown are the average of three independent experiments)

A

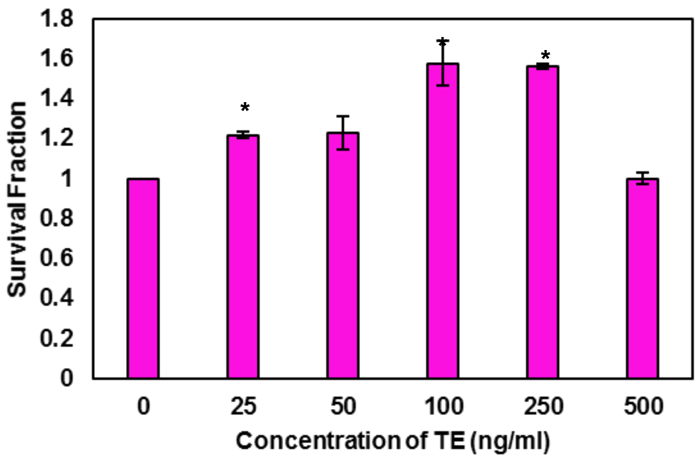

B

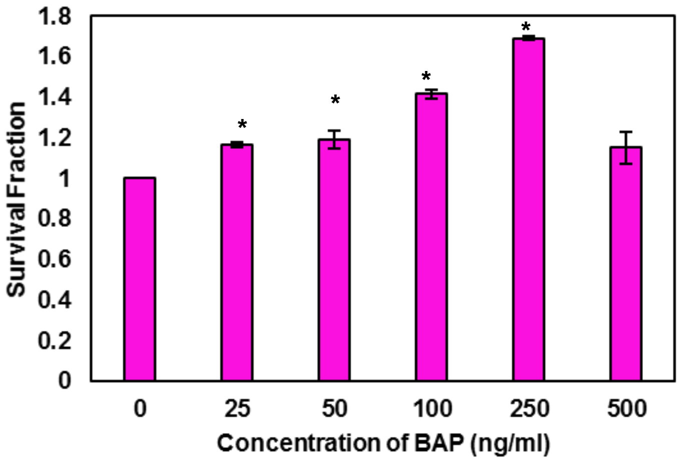

C

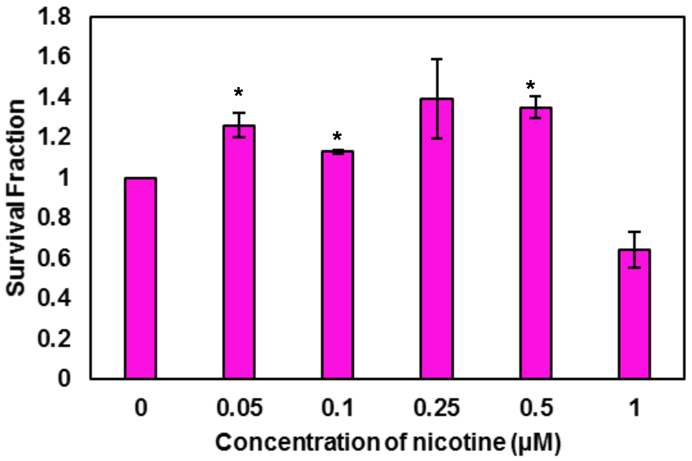

Figure S2: Clonogenic assay showing the survival efficiencies of SAS cells after treatment with TE, BAP, and nicotine. A, B, and C; Bar graph of clonogenic assay revealing the survival efficiencies of TE, BAP, and nicotine-treated SAS cells. Data are means ± SE *= $p < 0.05$.

A

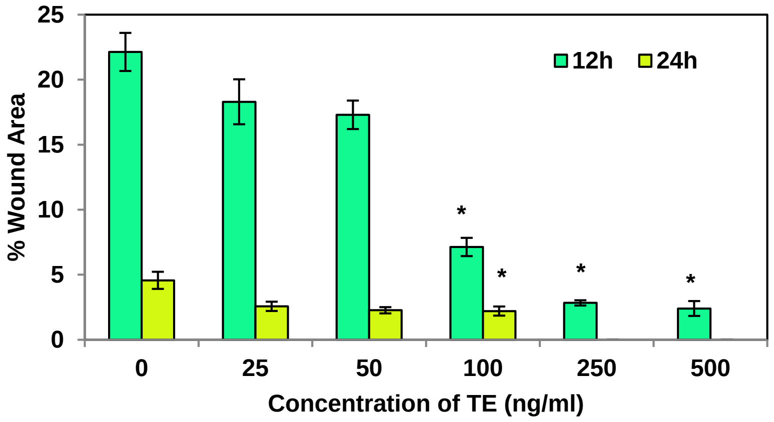

B

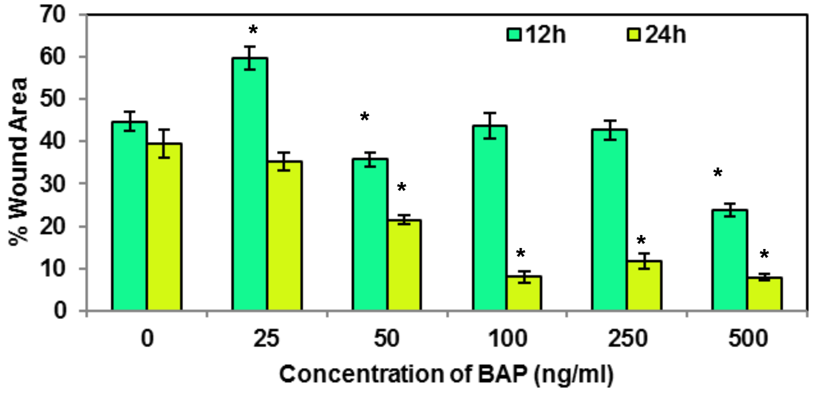

C

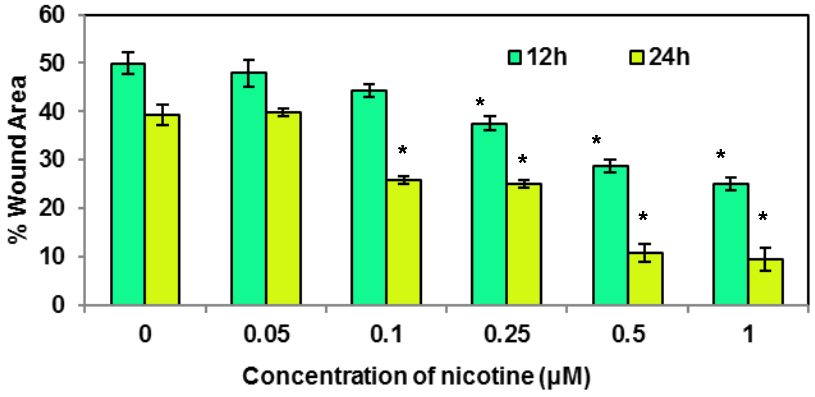

Figure S3: The treatment of TE, BAP and nicotine increases the directional cell migration as observed by the scratch wound healing assay. A, B, and C; Bar graph showing the % of cell-covered area (wound area) are shown in the bar diagram was estimated using the ImageJ software (1.510 Version). Data are means ± SE *= $p < 0.05$.
